# The Virtual Couch: a Curriculum on the Question of the Fundamentals of Remote Psychotherapy—Pilot Study

**DOI:** 10.1007/s40596-023-01805-6

**Published:** 2023-06-26

**Authors:** Julia Shekunov, Cosima Swintak, Kristin Somers, Bhanuprakash (Bhanu) Kolla, Anne Ruble, Seamus Bhatt-Mackin, David Topor, Aimee Murray, Magdalena Romanowicz

**Affiliations:** 1 Mayo Clinic, Rochester, MN USA; 2 Johns Hopkins University, Baltimore, MD USA; 3 Duke University, Durham, NC USA; 4 VA Boston Healthcare System, Boston, MA USA; 5 University of Minnesota, Minneapolis, MN USA

**Keywords:** Telehealth, Psychotherapy, Curriculum, Virtual, Telepsychiatry

## Abstract

**Objective:**

With a rise in remote clinical practice related to the COVID-19 pandemic, a novel remote psychotherapy curriculum was presented to psychiatry residents and fellows to address the urgent need to teach trainees how to adapt traditional psychotherapy skills to telepsychiatry settings.

**Methods:**

Trainees completed a survey before and after receiving the curriculum to assess remote psychotherapy skills and areas for growth.

**Results:**

Eighteen trainees (24% fellows, 77% residents) completed the pre-curriculum survey, and 28 trainees (26% fellows, 74% residents) completed the post-curriculum survey. Thirty-five percent of pre-curriculum participants indicated no experience with remote psychotherapy. Technology (24%) and patient engagement (29%) were identified as the greatest challenges in providing teletherapy pre-curriculum. Content related to patient care (69%) and technology (31%) was of most interest to pre-curriculum participants and identified as most helpful post-curriculum (53% and 26%, respectively). After receiving the curriculum, most trainees planned to make internal, provider-related changes to their remote teletherapy practice.

**Conclusions:**

The remote psychotherapy curriculum was well received by psychiatry trainees who had limited experience with remote clinical practice prior to the pandemic.

The COVID-19 pandemic prompted a practice change in psychiatry via a surge in remote clinical practice. Prior to the pandemic, only 15% of practices relied on remote clinical practice to interact with patients [1]. Consequently, most academic programs lacked robust curricula to teach the nuances of practicing medicine remotely [2]. Psychiatry is especially well-suited for implementing remote practice considering its long history of providing telehealth services [3]. However, many clinicians continue to acknowledge a lack of skills and confidence in their ability to provide telepsychiatry care [4]. Specifically, remote psychotherapy is defined here as therapy done with the help of video-based conferencing tools, something few trainees and few of their supervisors practiced or were taught during their training. Remote clinical practice is used here as a broader term that includes both remote psychotherapy and remote medication management. The COVID-19 pandemic introduced an urgent need to teach residents and fellows general remote psychotherapeutic skills. It highlighted the importance of teaching them how to utilize video-based conferencing tools to care for special populations, such as children and patients with psychosis, anxiety, or trauma [5].

As the world slowly begins to settle into the post-pandemic “new normal,” the authors believe that remote psychotherapy will continue to be offered to patients to ensure that they have uncomplicated and equitable access to high-quality psychiatric care. Thus, a need remains for a curriculum to educate residents and fellows on adapting traditional psychotherapy skills to telepsychiatry settings. For example, core therapeutic skills and main common factors such as therapeutic alliance with the patient, empathic listening, setting boundaries, and treatment goals will remain essential to telepsychiatry-delivered care and will require the development of a new set of skills that in our curriculum is referred to as “website manner” as well as “telepresence” [6–8].

To help trainees and supervisors understand their own skill sets and identify areas of growth in conducting remote psychotherapy, the authors developed a self-assessment survey that participants completed before and after the curriculum delivery. The authors hypothesized that the curriculum would facilitate trainees gaining skills in remote psychotherapy and inspire positive changes to their clinical practice.

## Methods

The authors prepared a single-group intervention with pre- and post-curriculum surveys. All psychiatry residents and child and adolescent psychiatry fellows at a single institution (*N* = 48) were recruited via an email from the investigators, which provided general information about the research study including that participation is voluntary and the research purpose, requirements (e.g., completing a remote training session and a pre- and post-curriculum survey), and key information, including remuneration. Participants received reimbursement of $25 upon completion of the study ($10 for pre-survey and $25 for post-survey).

All participants attended training on remote psychotherapy, which was delivered in a single, 60-min virtual session by Magdalena Romanowicz, M.D. (Mayo Clinic), Anne Ruble, M.D., M.P.H. (Johns Hopkins University), Seamus Bhatt-Mackin, M.D., FAPA, CGP (Duke University), David Topor, Ph.D., M.S. H.P.Ed. (VA Boston Healthcare System, Harvard Medical School), and Aimee Murray, Psy.D., LP (University of Minnesota) [9]. These colleagues hold leadership roles in education and have identified remote psychotherapy curricula as crucial to their residency and fellowship programs.

The curriculum comprised the following sections: introduction, highlighting the need for both trainees and supervisors to learn skills in remote psychotherapy; strategies for creating telepresence including orienting the patient to the virtual environment, awareness of the impact of emotions, maximizing aspects that enhance presence (such as lighting and position from camera, verbal versus nonverbal communication), minimizing distractions (such as fidgeting, sounds, pets, hiding own image on screen), and practicing via role-play to solidify skills (with video example); strategies for managing boundaries, including considerations about privacy, visit timing, and dress/appearance (with a video example of a small-group discussion of cases); strategies for building therapeutic alliance, including creating mini-goals, regularly checking-in about communication, making verbal that which is nonverbal when in person, speaking to shared experiences with technology (e.g., eye contact) as an example of process self-disclosure, predicting the possibility of technical “rupture,” and collaboratively developing a back-up communication plan; and remote psychotherapy in special populations, including discussion of absolute contraindications and considerations in patients with psychosis, anxiety, past trauma, and personality disorders, as well as children and adolescents.

Outcomes were assessed immediately pre- and post-curriculum via a qualitative and quantitative electronic survey created by the authors. Two research team members (MR and JS) coded the free-text responses in questions 5 through 8 by grouping responses into the most common categories. The pre-curriculum survey asked the following questions: (1) Are you a resident or a fellow? (2) How many years have you been in psychotherapy practice (include time in residency and fellowship)? (3) What percentage of your clinical practice is virtual? (4) What percentage of your pre-pandemic clinical practice was virtual? (5) Which concepts do you hope will be discussed in the course? (Responses were coded as patient care or technology.) (6) What aspects have been most surprising in your remote psychotherapy work? (Responses were coded as access to treatment, therapeutic process, or has not had experience.) (7) What aspects have been most beneficial in your remote psychotherapy work? (Responses were coded as access to treatment, therapeutic process, or has not had experience.) (8) What situations have been most challenging in your remote psychotherapy work? (Responses were coded as technology, patient engagement, or has not had experience.)

The post-curriculum survey asked the same first four questions as the pre-curriculum survey, as well as the following four different questions: (5) Which concepts discussed in the course will be the most helpful and productive in your practice? (Responses were coded as patient care or technology.) (6) Based on what you learned, what changes might you make to your video-based telehealth practice? (Responses were coded as patient-related, provider-related, or none/non-applicable.) (7) What steps do you anticipate needing to take to move toward the changes? (Responses were coded as internal, external, or none/non-applicable.) (8) What barriers do you anticipate in making these changes? (Responses were coded by the number of barriers identified.)

All participants were de-identified. All the responses were anonymized. No demographic information was obtained to ensure there was no possibility of identifying trainees. For statistical analysis, pre- vs. post-curriculum scores were contrasted using mixed effects analysis techniques (ANOVA for continuous variables; logit link function for dichotomous variables). This was a pilot study to determine the proof of concept; thus, power calculation could not be performed.

This study was approved by the Mayo Clinic Institutional Review Board (IRB) prior to the initiation of the project. Before submission to the IRB, the Education Research Committee approved the study.

## Results

Eighteen trainees (24% fellows, 77% residents) completed the pre-curriculum survey, and 28 trainees (26% fellows, 74% residents) completed the post-curriculum survey. Mean years in practice was 1.5 years (SD 1.37) for pre-curriculum participants and 2.2 years (SD 1.58) for post-curriculum participants. Pre-pandemic, pre-curriculum participants engaged in zero time in remote clinical practice; post-curriculum participants reported a mean of 3% (SD 3.55) time in remote practice pre-pandemic. The mean time spent in remote clinical practice at the time of the survey, during the COVID-19 pandemic, was 14% (SD 15.15) for pre-curriculum participants and 27% (SD 20.15) for post-curriculum participants. Thirty-five percent of pre-curriculum participants indicated no experience with remote psychotherapy.

Participants were most interested in content related to patient care (69%), including setting a frame, managing distractions in the patient’s environment, managing a crisis, and building rapport, and technology (31%), including video optimization, caring for patients across state lines, and completing examinations in virtual settings. In reflecting on their remote psychotherapy work, participants were most surprised by themes related to access (47%), including reduced no-show rates and patient preference for remote psychotherapy, and the therapeutic process (24%), including difficulties establishing rapport and benefits in seeing patients’ home environment. Access and the therapeutic process were aspects also identified as most beneficial in participants’ remote work (47% and 18% respectively). Technology (24%) and patient engagement (29%) were identified as the greatest challenges (Table 1).

**Table 1:** Examples of quotations from psychiatry residents and child and adolescent psychiatry fellows (pre-curriculum)

Question	Theme	Quote
Q1. Which concepts do you hope will be discussed in the course?	Patient	Setting a frame/boundaries; how to manage distractions/events occurring in the patient’s home/environment
	Technology	Mental status examination in virtual settings; creating a professional virtual space; setting up virtual patient panels; caring for patients in multiple states across the country
Q2. What aspects have been most surprising in your remote psychotherapy work?	Access	The remote psychotherapy sessions I have participated in as a medical student were largely with adolescents, but I was surprised by how many kids opted for this type of therapy even when it was feasible for them to come into clinic
	Therapeutic process	The rapport/interaction feels more distant compared to in person
Q3. What aspects have been most beneficial in your remote psychotherapy work?	Access	Greater accessibility for the patient; able to see full face (as opposed to masked when in office)
	Therapeutic process	At times, patients are more comfortable going into more difficult material, likely because of the safety of the home environment
Q4. What situations have been most challenging in your remote psychotherapy work?	Technology	Technological disruptions create distance
	Patient engagement	Patients often more distracted, especially children and adolescents. I also really miss coworkers and colleagues while doing remote work

In the post-curriculum survey, the most helpful concepts in the presentation were related to patient care (53%) and technology (26%). Most participants (79%) indicated that they might make provider-related changes to their remote telehealth practice based on what they learned and anticipated needing to take internal steps (58%), defined by the authors as changes physicians can make using their own resources, or internal resources, such as developing a plan for orienting patients to the virtual space, outlining a safety conversation, and incorporating strategies into setting a frame, compared to external (16%) to move toward the changes. External steps, defined here as involving resources such as other people or additional tools, include meeting with colleagues to calibrate remote setup, and discussing content of training with supervisors. Participants identified no anticipated barriers to making these changes (32%), one barrier (26%), two barriers (37%), and three barriers (5%) (Table 2).

**Table 2:** Examples of quotations from psychiatry residents and child and adolescent psychiatry fellows (post-curriculum)

Question	Theme	Quote
Q1. Which concepts discussed in the course will be the most helpful and productive in your practice?	Patient	It was very helpful to learn how to prepare patients for virtual visits and to hear some of the evidence related to patient satisfaction for virtual visits
	Technology	Setting frame with video visits, addressing technological concerns like mentioning apparent lack of eye contact with video camera, etc
Q2. Based on what you learned, what changes might you make to your video-based telehealth practice?	Patient	Set more formal boundaries/expectations
	Provider	Calling to our attention the oddities of speaking on a camera, verbally expressing what is happening rather than relying on their eyesight
Q3. What steps do you anticipate needing to take to move toward the changes?	Internal	Develop an outline of how to orient patients to the virtual space
	External	Educational time allocated to work on it
Q4. What barriers do you anticipate in making these changes?	List the barriers	Not having enough practice in implementing them and getting comfortable using them That I am new in training and not experienced in telehealth

Pre-curriculum themes included trainees wanting to learn about setting a frame and boundaries, and managing distractions occurring in a patient’s home. Many also mentioned that they struggled with performing mental status examinations in virtual settings (including how to assess body language). Several trainees indicated they wanted to learn about the legal aspects of caring for patients living or traveling across state lines. Lastly, trainees were concerned about the therapeutic alliance and being able to connect with patients via the internet. Trainees who had already tried virtual therapy felt that the rapport was more distant than in-person therapy. At the same time, several of the trainees mentioned that they learned more about their patients by studying their home environments and backgrounds. They also liked that the no-show rate decreased because virtual sessions were more accessible. Trainees thought the most beneficial aspects of virtual psychotherapy were its accessibility and psychological safety of the environment. The most challenging aspects of virtual psychotherapy were difficulties with communication, distractions in the house, and lack of privacy.

Post-curriculum themes appreciated by trainees included learning to set a frame and boundaries. They also appreciated that the curriculum included how to adjust interview techniques to optimize the experience while still obtaining an accurate clinical picture of the patient. After receiving the curriculum, trainees planned to start therapy with their patients by discussing frame and boundaries. Many decided to make a script introducing patients to video therapy to make it easier for both parties to adjust. Trainees planned to ask patients about the lighting and camera setup and discuss safety in the virtual space. Barriers to implementation of skills included not having enough time to practice strategies that they had learned during the training, internet access, connectivity issues, being able to recall the script that they created, lack of experience, and patient preferences.

## Discussion

To our knowledge, our study represents the first attempt to create an easy-to-implement remote psychotherapy curriculum for psychiatry residents and child and adolescent psychiatry fellows. Although trainees are already familiar with navigating the use of technology, they report needing help with establishing boundaries and setting a frame in remote psychotherapy.

One of the challenges of this study was to create pre- and post-curriculum surveys and appropriately code the responses. The decision was made to use both a qualitative and quantitative approach to best summarize the trainees’ experiences and perspectives.

Pre-pandemic, pre-curriculum participants engaged in zero time in remote clinical practice (defined as time in both remote psychotherapy and remote medication management); post-curriculum participants continued to report a very small percentage of their time engaging in remote clinical practice. This is consistent with other studies showing that despite advancements in technology, remote clinical practice is still largely underused. In 2009, only around 2% of psychiatrists reported its use, which did not increase substantially in the following years [10]. In our study, about one-third of trainees reported no experience with remote psychotherapy specifically, which underscores the importance of remote psychotherapy training. The results of our virtual curriculum discussed in this brief report are promising. We hope they will guide other programs on how to implement teaching on remote psychotherapy that could improve trainees’ confidence in providing services in the virtual environment. Ideally, a curriculum should be followed by supervision with experienced therapists who practice virtual therapy and could provide ongoing guidance to the trainees, which could be a barrier in some programs, where not all faculty feel comfortable with providing tele-services.

There are important limitations to this study. Results do not permit strong causal attributions due to the small sample size and single-group, pre-post study design. There may be a learning effect from the pre- and post-curriculums.

In conclusion, the response among psychiatry residents and fellows was favorable for delivery of a well-rounded curriculum on remote psychotherapy. Participants had limited experience with remote clinical practice prior to the COVID-19 pandemic. Access and the therapeutic process were identified as the most surprising and beneficial aspects to participants in their current remote psychotherapy work. Technology and patient engagement were identified as the biggest challenges. Participants were particularly interested in learning how to best utilize technology and adapt principles of clinical care to the virtual space. After completing the curriculum, participants indicated that the content on patient care and use of technology was most beneficial. The majority planned to make internal provider-related changes in optimizing their remote clinical practice.
